# *De Novo* Transcriptome and Expression Profile Analysis to Reveal Genes and Pathways Potentially Involved in Cantharidin Biosynthesis in the Blister Beetle *Mylabris cichorii*

**DOI:** 10.1371/journal.pone.0146953

**Published:** 2016-01-11

**Authors:** Yi Huang, Zhongkang Wang, Shenfang Zha, Yu Wang, Wei Jiang, Yufeng Liao, Zhangyong Song, Zhaoran Qi, Youping Yin

**Affiliations:** 1 Key Laboratory of Genetic Function and Regulation, School of Life Science, Chongqing University, Chongqing 400030, China; 2 Panzhihua University, Panzhihua 617000, China; 3 Clinical Medicine college of Tianjin Medical University, Tianjin 300270, China; University Paris South, FRANCE

## Abstract

The dried body of *Mylabris cichorii* is well-known Chinese traditional medicine. The sesquiterpenoid cantharidin, which is secreted mostly by adult male beetles, has recently been used as an anti-cancer drug. However, little is known about the mechanisms of cantharidin biosynthesis. Furthermore, there is currently no genomic or transcriptomic information for *M*. *cichorii*. In this study, we performed *de novo* assembly transcriptome of *M*. *cichorii* using the Illumina Hiseq2000. A single run produced 9.19 Gb of clean nucleotides comprising 29,247 sequences, including 23,739 annotated sequences (about 81%). We also constructed two expression profile libraries (20–25 day-old adult males and 20–25 day-old adult females) and discovered 2,465 significantly differentially-expressed genes. Putative genes and pathways involved in the biosynthesis of cantharidin were then characterized. We also found that cantharidin biosynthesis in *M*. *cichorii* might only occur via the mevalonate (MVA) pathway, not via the methylerythritol 4-phosphate/deoxyxylulose 5-phosphate (MEP/DOXP) pathway or a mixture of these. Besides, we considered that cantharidin biosynthesis might be related to the juvenile hormone (JH) biosynthesis or degradation. The results of transcriptome and expression profiling analysis provide a comprehensive sequence resource for *M*. *cichorii* that could facilitate the in-depth study of candidate genes and pathways involved in cantharidin biosynthesis, and may thus help to improve our understanding of the mechanisms of cantharidin biosynthesis in blister beetles.

## Introduction

The blister beetle *Mylabris cichorii* (Coleoptera: Meloidae), has been widely used to treat a variety of conditions such as rabies, dropsy, warts, impotence and fever [1]. The dried body of *M*. *cichorii* has been used in traditional Chinese medicine for thousands of years [2], and the preparation was also recorded in the Chinese Pharmacopoeia [3]. Cantharidin is a sesquiterpenoid, synthesized as a defensive substance, by most adult blister beetles when they are attacked [4]. Cantharidin and its derivatives have recently been used to treat several cancers, including liver, lung, esophageal, and stomach cancers [5,6], as well as other diseases [7]. Moreover, Cantharidin is a feeding deterrent to most insects and also has some fungicidal and herbicidal properties [8,9].

Over the past few decades, studies of blister beetles have focused mainly on their biology and ecology, artificial breeding technologies, and antitumor mechanisms [4,7,10,11]. Although some studies have reported cantharidin biosynthesis [12–18], the pathways involved in this process in meloid beetles remain poorly understood. As of October 2015, only 12 nucleotide sequences and 135 expressed sequence tags (ESTs) for *M*. *cichorii* were deposited in the National Center for Biotechnology Information (NCBI) database.

Previous research revealed that most blister beetles demonstrate sexual dimorphism in terms of cantharidin production. Cantharidin is mostly synthesized by adult male beetles and transferred to adult females as a precopulatory gift [4,10]. Cantharidin level in male *M*. *cichorii* was low right after emerging as adult, but dramatically increased within 20-25days, and peaked at 30 days after adult emergence. In contrast, cantharidin levels remained low in females [19].

In the present study, we conducted *de novo* transcriptome and expression profiling analysis of *M*. *cichorii* using an Illumina HiSeq 2000 platform to gain a deeper insight into the genes involved in cantharidin biosynthesis. Besides, the assembled, annotated transcriptome sequences and gene expression profiles analysis will provide an invaluable resource for future studies on blister beetles.

## Materials and Methods

### Insect specimens

**Fig 1:**
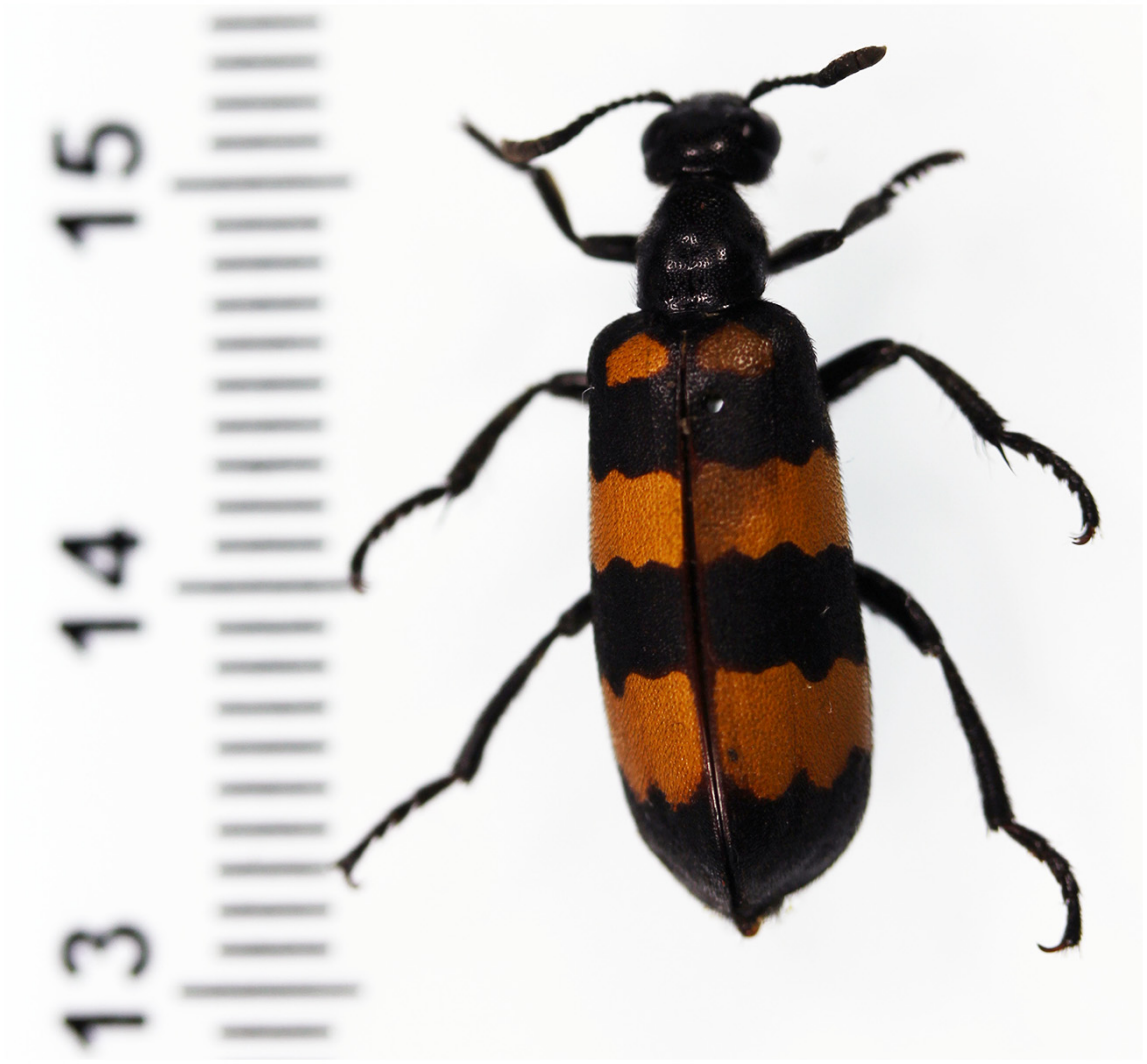
The blister beetle *M*. *cichorii*.

*M*. *cichorii* adults (Fig 1) were originally collected from Luodian, Guizhou Province of China and were reared in our laboratory with a semiartificial diet (ddH_2_O, 80 ml; soybean powder, 15 g; yeast powder, 9 g; *Luffa acutangula* flower powder, 7.5 g; glucose, 7.5 g; milk powder, 3 g; *Flos Sophorae* flower honey, 3 ml; agar, 2 g; and sorbic acid, 0.3 g) at 30 ± 1°C, 75 ± 5% relative humidity, and a photoperiod of 14 h light: 10 h dark in a dedicated environmentally controlled chamber. Female adults laid eggs in soil and eggs were maintained under similar conditions for hatching. Each larva was reared with one *Locusta migratoria manilensis* egg mass in a plastic drinking cup filled with soil [19]. Adults used for transcriptome and expression profile sequencing were first generation adults reared sex-segregate under the conditions mentioned above. For transcriptome sequencing, 10 pooled samples: 1–5, 7–10, 12–15, 17–19, and 20–25 day-old (3 males and 3 females biological replication sampled at each of every single day) were collected. For expression profile sequencing, 2 separate samples: 20–25 day-old (3 males and 3 females biological replication sampled at each of every single day) were collected. RNA was immediately extracted from the fresh samples.

### RNA isolation and cDNA library preparation for *de novo* transcriptome analysis

Total RNA was isolated using TRIzol reagent (Invitrogen, USA) according to the manufacturer’s instructions. Total RNA samples were digested using DNase I to remove potential genomic DNA. RNA quality and yield were assessed using a 2100 Bioanalyzer (Agilent Technologies, USA). Poly(A)^+^ RNA was purified from 10 μg pooled total RNA (1 μg per sample) using oligo(dT) magnetic beads according to Illumina manufacturer’s instructions, and mixed with the fragmentation buffer. The mRNA was then fragmented into short sequences (about 200 bp) in the presence of divalent cations under high temperature. The cleaved RNA fragments were used for first-strand cDNA synthesis using reverse transcriptase and random primers, followed by second-strand cDNA synthesis using DNA polymerase I and RNaseH. After end repair, 3'-end single nucleotide A (adenine) addition and ligation of adaptors, the products were purified and amplified by PCR to create the final cDNA library. The quality and quantity of the sample library was assured using an Agilent 2100 Bioanalyzer and ABI StepOnePlus Real-Time PCR System (Life Technologies, USA).

### *De novo* assembly of Illumina sequencing results

The sequencing library was sequenced using the Illumina Hiseq2000 platform. The raw reads were filtered to obtain high-quality *de novo* transcriptome sequence data. All reads with adaptor contamination, unknown nucleotides comprising more than 5%, and low-quality reads (>20% base with quality value ≤10 in a read) were discarded. *De novo* transcriptome assembly of these short reads was carried out using the short-read assembling program Trinity [20]. The program initially formed contigs representing the significant parts of individual isoforms. The contigs were then formed into clusters and complete de Bruijn graphs were produced for each cluster, representing the full transcriptional complexity for a given gene (or set of genes that share sequences in common), and the full read set was partitioned among these disjoint graphs. In addition, Trinity processes the individual graphs in parallel, tracing the paths that reads and pairs of reads take within the graph, ultimately reporting full-length transcripts for alternatively-spliced isoforms, and teasing apart transcripts that correspond to paralogous genes. The resulting sequences were referred to as unigenes.

### Unigene function annotation and classification

To annotate the transcriptome, we performed a BLAST search against the non-redundant NCBI nucleotide database Nt, protein database Nr, SwissProt, KEGG, and COG with an E-value threshold of 1.0E-5, retrieving proteins with the highest sequence similarities with the given unigenes, together with their protein functional annotations. GO annotations were assigned by Blast2GO [21] by searching the Nr database (http://www.blast2go.com/b2ghome). After acquiring the GO annotation for every unigene, we used WEGO software [22] to carry out GO functional classification for all unigenes and to determine the distributions of gene functions of the species at the macro level (http://wego.genomics.org.cn/). KEGG pathway annotations were carried out using Path_finder software against the KEGG (http://www.genome.jp/kegg/) database [23].

### Expression profile library construction and analysis

Expression profile libraries were prepared in parallel for the male and female *M*. *cichorii* samples (20–25 days after adult emergence; 1 μg total RNA per sample) using the same methods as for the *M*. *cichorii de novo* transcriptome library construction. The library products were then sequenced via Illumina HiSeq 2000. All the sequences were filtered to remove adaptor reads, unknown nucleotides comprising more than 10%, low-quality reads (>50% bases with quality value ≤5) to obtain clean reads. Clean reads were then mapped to reference sequences (*M*. *cichorii de novo* transcriptome) using SOAPaligner/SOAP2 software [24], allowing no more than two nucleotide mismatches.

The expression level for each gene was determined by the numbers of reads uniquely mapped to the specific gene and the total number of uniquely-mapped reads in the sample, and calculated using the RPKM method [25] (reads per kb per million reads).

DEGs between males and females were screened according to a strict algorithm based on Audic and Claverie’s [26] method. If the number of unambiguous clean tags from gene A is x, given that every gene’s expression occupies only a small part of the library, p(x) will closely follow the Poisson distribution.

$$\text{p}\left(\text{x}\right)=\frac{e^{-\lambda }\lambda^{x}}{x!}$$

where λ is the real transcripts of the gene.

The total clean tag number of the sample 1 is N1, and of sample 2 is N2; gene A holds x tags in sample 1 and y tags in sample 2. The probability of gene A being expressed equally between the two samples can thus be calculated as:

$$2\overset{i=y}{\underset{i=0}{\sum }}p\left(i\;|x\right)$$

$$\text{Or}2\times \left(1-\Sigma_{i=0}^{i=y}\;p\left(i\;|x\right)\right)\;(\text{if }\Sigma_{i=0}^{i=y}\;p\left(i\;|x\right)>0.5)$$

$$\text{p}\left(\text{y}|\text{x}\right)={\left(\frac{N_{2}}{N_{1}}\right)}^{y}\frac{\left(x+y\right)!}{x!y!{\left(1+\frac{N_{2}}{N_{1}}\right)}^{\left(x+y+1\right)}}$$

The P-value corresponds to the differential gene expression test. The false discovery rate (FDR) is used to determine the threshold of the P-value in multiple tests [27]. An FDR ≤0.001 and absolute value of log2ratio ≥1" were used as the threshold to determine the significance of differential gene expression.

The DEGs were used for GO functional enrichment analysis and KEGG pathway enrichment analysis. GO enrichment analysis provides all GO terms that are significantly enriched in DEGs compared with the transcriptome background, and filters out DEGs that correspond to biological functions. We first mapped all DEGs to GO terms in the database (http://www.geneontology.org/), calculated the gene numbers for every term, then used hypergeometric tests to identify significantly-enriched GO terms in DEGs compared with the transcriptome background, according to the formula:

$$\text{P}=1-\overset{m-1}{\underset{i=0}{\sum }}\frac{\left(\begin{array}{c} M\\ i \end{array}\right)\left(\begin{array}{c} N-M\\ n-i \end{array}\right)}{\left(\begin{array}{c} N\\ n \end{array}\right)}$$

In this equation, N represents the number of genes with GO annotation; n represents the number of DEGs in N; M represents the number of genes that are annotated to the certain GO terms; and m represents the number of DEGs in M. For GO enrichment analysis, all the P-values were obtained by Bonferroni correction. We selected a corrected P-value ≤0.05 as a threshold to determine significantly-enriched GO terms in DEGs. KEGG pathway enrichment analysis identifies significantly-enriched metabolic pathways or signal transduction pathways in DEGs compared with the background reference sequences. The calculating formula is the same as for GO analysis, where N is the number of all genes with KEGG annotation, n is the number of DEGs in N, M is the number of all genes annotated to specific pathways, and m is number of DEGs in M. Q-value ≤0.05 was selected as a threshold to determine significant enrichment of DEGs.

### RT-qPCR validation of DEGs

A total of 13 representative genes that were prominently and differentially expressed in our expression profile data were chosen for validation by RT-qPCR. Total RNA was extracted as described for *de novo* transcriptome and expression profile library preparation, treated with DNase I (Thermo Scientific, USA), and reverse transcribed to cDNA using RevertAid First Strand cDNA Synthesis Kit (Thermo Scientific, USA). The quantitative reaction was performed on a CFX96 Real-Time PCR Detection System (Bio-Rad, USA) using GoTaq qPCR Master Mix (Promega, USA). The RT-qPCR reaction was run at 95°C for 3 min followed by 40 cycles at 95°C for 10 s, 58°C for 20 s, and at 72°C for 15 s. The reaction melting-curve analysis from 65°C to 95°C was then applied to all reactions to ensure consistency and specificity of the amplified product. Three biological duplicates of each sample and triplicates of each reaction were acquired. The results were normalized to the expression level of the internal reference genes *UBE3A* (ubiquitin-protein ligase E3A) and *RPL22e* (ribosomal protein) [28]. All primers used in this study are listed in S1 Table. The expression ratios were calculated from cycle threshold values using the 2^−ΔΔCT^ method.

## Results and Discussion

### Illumina sequencing and *de novo* assembly

**Fig 2:**
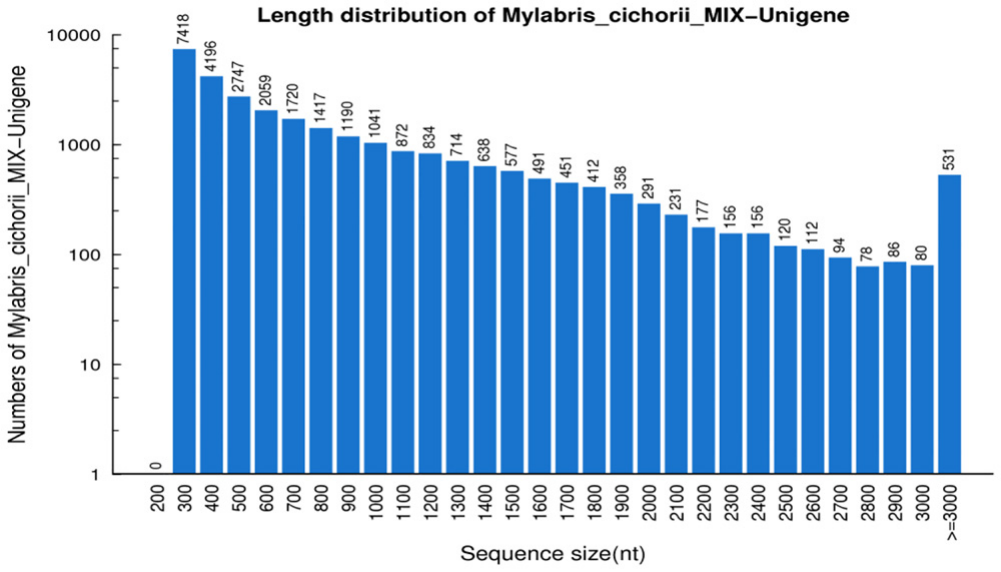
Length distribution of assembled unigenes. The number of y-axis has been transfer into logarithmic scale.

We sequenced the transcriptome of *M*. *cichorii* using Illumina high-throughput sequencing technology and obtained 9.19 Gb of coverage, with 109,674,770 clean reads. After removing adaptor sequence, ambiguous nucleotides, and low-quality sequences, 109,674,770 clean reads with an average length of 91 bp remained. The clean reads were then assembled into 49,923 contigs and the contigs were clustered together to produce 29,247 unigenes with a mean length of 788 bp and an N50 of 1185 bp (Table 1). The length statistics of the assembled unigenes are displayed in Fig 2. The sequencing raw data has been deposited into the Short Reads Archive (SRA) database under the accession number SRR1996329.

**Table 1:** Summary of sequence assembly after Illumina sequencing.

Description	Number
**Before trimming**	
Raw reads	124,866,282
**After trimming**	
Clean reads	109,674,770
Average length of clean reads (bp)	91
Q20 percentage	98.07
N percentage	0
GC content (%)	41.09
**After assembly**	
Total contigs	49,923
Mean length of contigs (bp)	420
N50 (bp) of contigs	807
Total unigenes	29,247
Mean length of unigenes (bp)	788
N50 (bp) of unigenes	1185

### Functional annotation and pathway assignment

**Fig 3:**
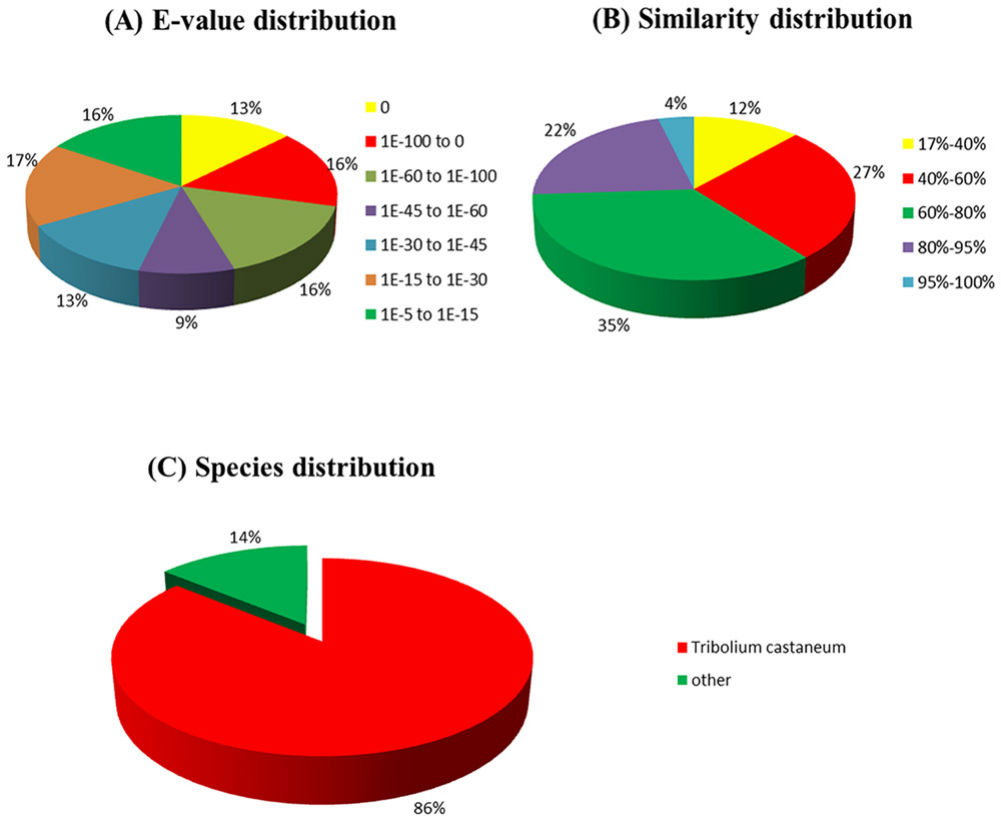
Homology search of Illumina sequences against the Nr database. **(A)** E-value distribution of BLAST hits for each unique sequence with a cut-off E-value of 1.0E^-5^. **(B)** Species distribution of the top BLAST hits for each sequence. **(C)** Species distribution as a percentage of the total homologous sequences with an E-value ≥1.0E^-5^. The first hit of each sequence was used for analysis.

Annotation provides expression information and functional annotation of unigenes, based on sequence similarity searches against public databases. Functional annotation information includes protein functional annotation, COG functional annotation, Gene Ontology (GO) functional annotation, and Kyoto Encyclopedia of Genes and Genomes pathway database (KEGG) information. For homology annotation, sequences were compared with protein databases including NCBI Nr, SwissProt, KEGG, and COG to detect similarities using blastx (E-value <10^−5^) and the nucleotide database NCBI Nt by blastn (E-value <10^−5^), and protein functions with the highest sequence similarity were retrieved. Finally, 23,739 unigenes (81.17%) were annotated. Comparison with the Nr, Nt, and Swissprot databases identified 23,201 (79.33%), 12,295 (42.04%), and 18,646 (63.75%) sequences, respectively. The E-value distribution of the top hits in the Nr database showed that 54% of the mapped sequences had strong homology (<1.0E^-45^), whereas 46% of the homologous sequences ranged from 1.0E^-5^–1.0E^-45^ (Fig 3A). The similarity distribution showed a comparable pattern, with 26% of the sequences having a similarity >80%, and 74% of the hits having similarities of 17–80% (Fig 3B). The species distribution of the best match result for each sequence showed that 86% of *M*. *cichorii* sequences matched with sequences from the model Coleopteran *Tribolium castaneum*, with a relatively low proportion of matches to other insects (Fig 3C).

**Fig 4:**
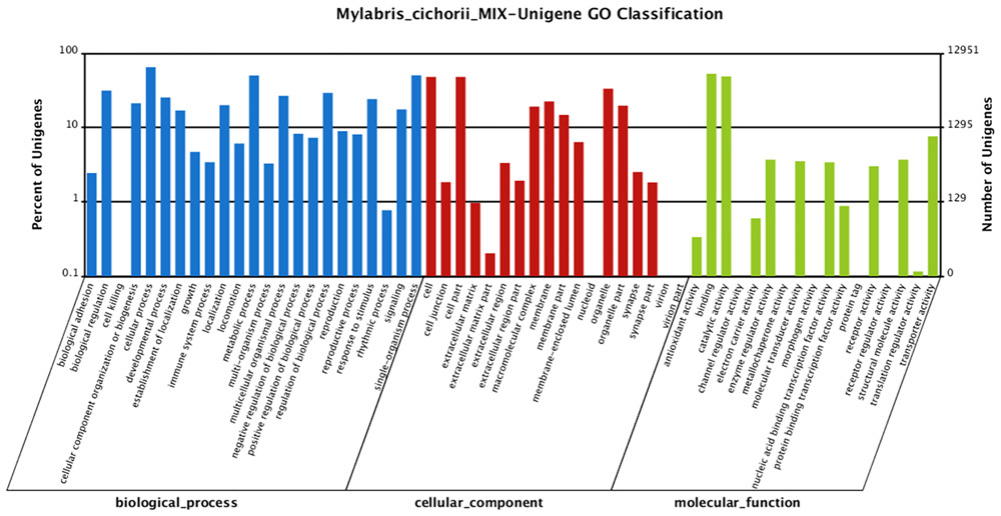
Gene Ontology analysis of genes in the Illumina *de novo* transcriptome of *M*. *cichorii*. The results are summarized in three main categories: biological process, cellular component, and molecular function. The righty-axis indicates the number of genes in a category. The left y-axis indicates the percentage of a specific category of genes in that main category.

Based on GO classifications, 12,951 (53.04%) sequences were classified into three major functional categories (biological processes, molecular functions, and cellular components) and 58 subcategories. Of these, the dominant terms were “cellular process”, “metabolic process”, “single-organism process”, “cell”, “cell part”, and “binding”, while few genes were classified into “cell killing”, “nucleoid”, and “channel regulator activity” terms (Fig 4).

**Fig 5:**
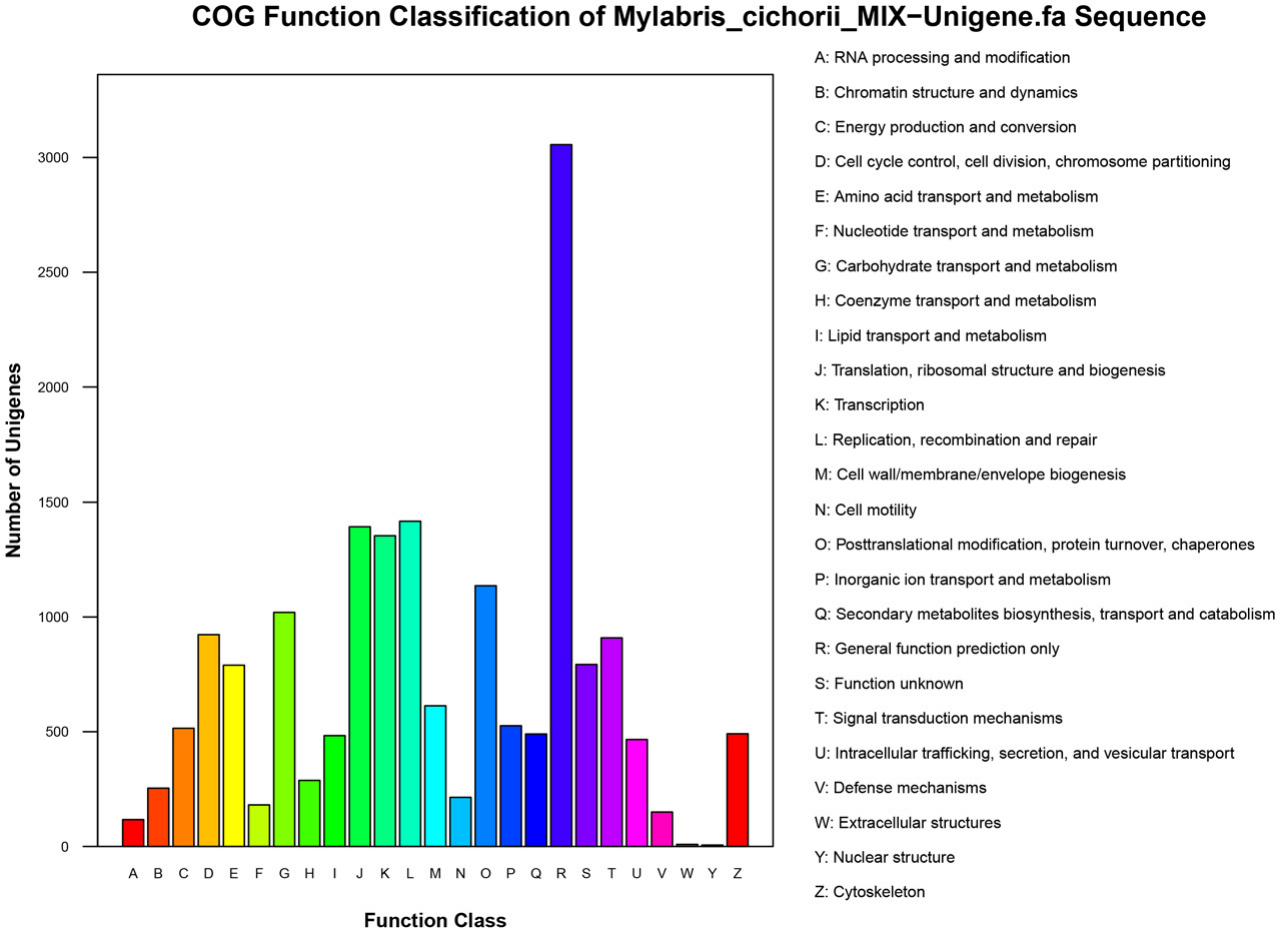
COG functional annotation of putative proteins. Of 23,739 Nr hits, 8,580 sequences had COG classifications among the 25 categories. The y-axis indicates the number of sequences in a category.

To classify orthologous gene products, 8,580 (36.14%) non-redundant unigenes were subdivided into 25 COG classifications. Among these, the cluster of “general function prediction only” (3,056; 35.62%) represented the largest group, followed by “replication, recombination and repair” (1,417; 16.52%), “translation, ribosomal structure and biogenesis” (1,393; 16.24%), and “transcription” (1,354; 15.78%), whereas “nuclear structure” (6; 0.0007%) comprised the smallest group (Fig 5).

Using KEGG, 16,660 unigenes (70.18%) were assigned to six specific pathways (metabolism, cellular processes, organism system, human diseases, genetic information processing, and environmental information processing) and 258 subcategories. The top five subcategories of pathways were “metabolic pathways” (2,285; 13.72%), “pathways in cancer” (577; 3.46%), “regulation of actin cytoskeleton” (577; 3.46%), “RNA transport” (530; 3.18%) and “focal adhesion” (502; 3.01%). “Lysine biosynthesis” only contained three members (0.02%).

### Expression profile library sequencing and analysis

Expression profiling was performed to give insight to the genes involved in cantharidin biosynthesis by identifying the differentially-expressed genes (DEGs) between 20–25 day-old adult males and females groups. After filtering, a total of 6,095,272 and 6,025,355 clean reads were obtained, respectively. The percentages of clean reads among the raw reads in the two libraries were 99.65% and 99.67%, respectively. The sequencing raw data has been deposited into the SRA database under the accession number SRR2014710 (20–25 day-old male adult) and SRR2014711 (20–25 day-old female adult). Gene annotation was performed by read-mapping analysis using the non-redundant consensus sequences from the reference database generated by the above *de novo* transcriptome sequencing. Results showed that 90.85% and 90.20%, respectively, of all the clean reads could be mapped to the reference database.

The expression level for each gene was calculated and displayed by an RPKM numerical value. The algorithm developed by Audic and Claverie [26] was then used to identify DEGs between the two groups. There were 2,465 genes with significantly different expression levels between the male and female libraries. Compared with females, 1,468 were up-regulated and 997 genes were down-regulated, respectively in males. Regarding 10 of the most-differentially up-regulated genes, only four had defined functions: protamine, a gene related to spermatid nucleus differentiation, two trypsin genes with catalytic activity, and a keratin-associated protein gene. Five of the 10 most-differentially down-regulated genes had defined functions: two serine protease family genes (chymotrypsin precursor and serine protease 120 precursor genes), a mucin gene, and a secretory eggshell protein precursor gene. Additionally, 11 genes among the 20 DEGs had unknown functions or no annotations (S2 Table). According to the GO classification, the DEGs were categorized into three major groups and 53 subgroups (S1 Fig), with “catalytic activity”, “metabolic process”, “cell”, “cell part”, and “binding” being the dominant terms. Meanwhile, 145 gene sets were significantly enriched, with “catalytic activity”, “metabolic process”, and “cytosolic ribosome” being the most-represented terms. To clarify the functions of the DEGs, all the genes were mapped to the terms in the KEGG database and compared with the whole transcriptome background. A total of 1,506 DEGs were assigned to 249 KEGG pathways, all of which were significant enrichment pathways (Q value ≤0.05).

### Identification of putative genes and pathways related to cantharidin biosynthesis

**Fig 6:**
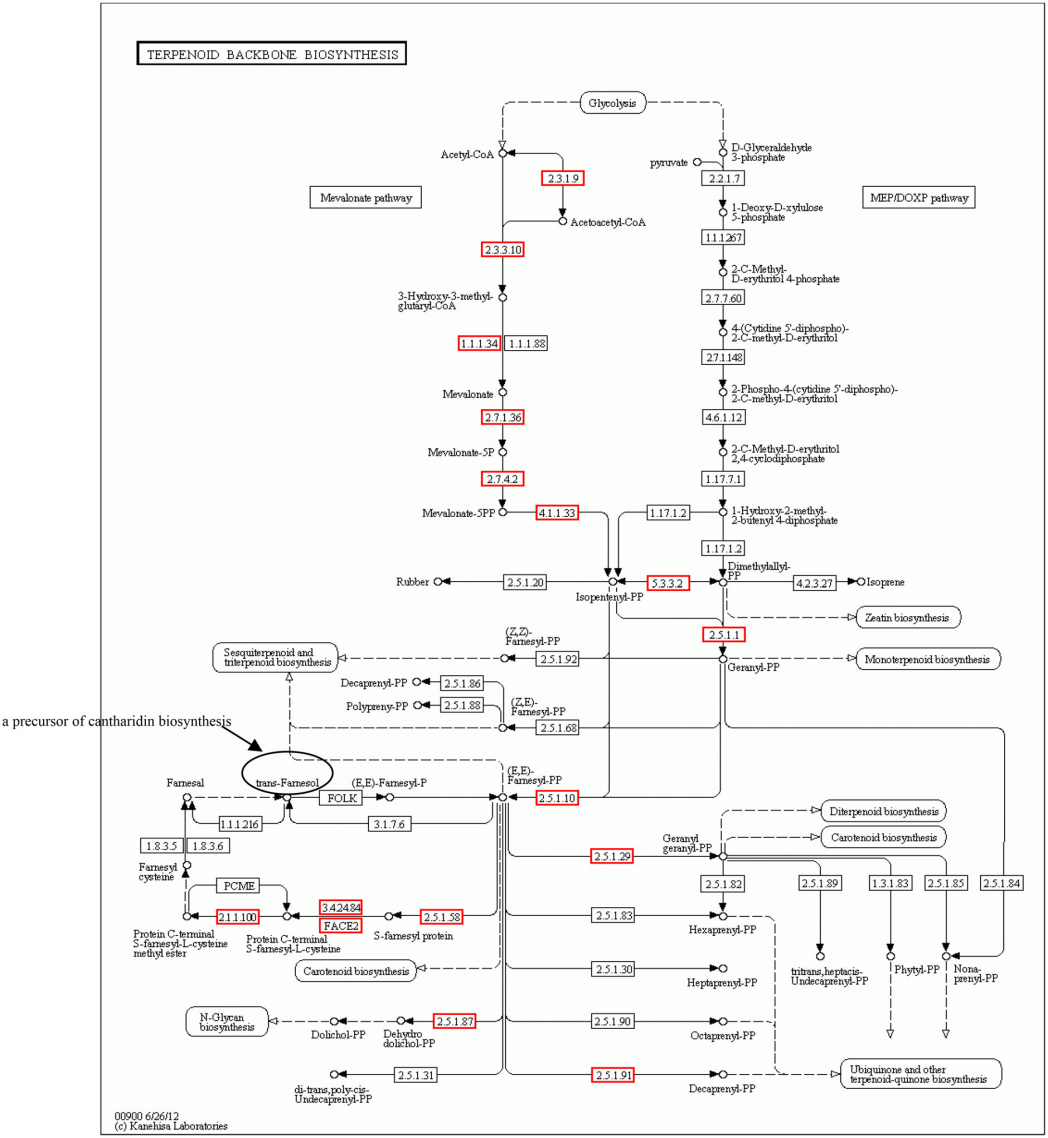
“Terpenoid backbone biosynthesis” pathway of *M*. *cichorii de novo* transcriptome. The red box indicates the homologous enzyme of the unigenes have been found in the transcriptome databases of *M*. *cichorii*, while the white box represents there is no unigene has been found.

Terpenoids are among the largest and the most structurally and functionally diverse groups of natural products, with many significant biological functions and economic values. Plants use two pathways to synthesize terpenoids: the mevalonate (MVA) pathway and the methylerythritol 4-phosphate/deoxyxylulose 5-phosphate (MEP/DOXP) pathway. The MEP/DOXP pathway has also been found in bacteria, algae, and the malaria protozoa, Plasmodium. However, previous studies did not indicate the existence of the MEP/DOXP pathway in insects [29–31]. It was not known whether the biosynthesis of sesquiterpenoid cantharidin in blister beetles occurs via MVA or MEP pathways [32]. We examined the *M*. *cichorii* unigenes in the “terpenoid backbone biosynthesis” pathway (map00900) and found that these were only distributed in the MVA pathway, with no unigenes in the MEP/DOXP pathway (Fig 6). All the enzymes of the MVA pathway were found in *M*. *cichorii*, including gacetyl-CoA C-acetyltransferase (*atoB*; 2.3.1.9), hydroxyl-methylglutaryl-CoA synthase (*HMGS*; 2.3.3.10), hydroxyl-methylglutaryl-CoA reductase (*HMGR*; 1.1.1.34), mevalonate kinase (*mvaK1*; 2.7.1.36), phosphomevalonate kinase (*mvaK2*; 2.7.4.2), and diphosphomevalonate decarboxylase (*MVD*; 4.1.1.33), suggesting that cantharidin biosynthesis in *M*. *cichorii* might only occurs via the MVA, and not the MEP pathway or a combination of these pathways. We also performed expression profiling analysis of the MVA pathway and found that *HMGS* (unigene 496) and *HMGR* (CL2009. Contig1) were significantly up-regulated in male beetles compared to female beetles. *HMGR* is considered to be a rate-limiting enzyme in the MVA pathway in higher plants [33–35]. Notably two unigenes were annotated as *atoB* gene, one of them (unigene5706) was down-regulated, while another (CL951. Contig3) was up-regulated in male beetles, suggesting that the *atoB* gene might play an isozyme role in the terpenoid biosynthesis pathway. Meanwhile, no gene was included in the “sesquiterpenoid and triterpenoid biosynthesis” pathway (map00909).

Farnesyl diphosphate (FPP) is the precursor of sesquiterpenoids [36,37]. We identified the key enzyme gene farnesyl diphosphate synthase (*FPPS*; 2.5.1.1) in our library and found that it was significantly up-regulated (unigene16392) in male compared with female beetles, while the gene for isopentenyl-diphosphate delta-isomerase (*IDI*; 5.3.3.2), another important enzyme in terpenoid biosynthesis, was also significantly up-regulated (CL3032. Contig1) in males.

A prior isotopic tracer study demonstrated that some of the hydrogen atoms in cantharidin were derived from trans-farnesol, implicating trans-farnesol as a precursor of cantharidin biosynthesis [18]. In other words, the upstream MVA pathway, gene *FPPS*, *IDI* are all related to the cantharidin biosynthesis. We also detected the genes belonging to the branched chain of trans-farnesol biosynthesis and discovered four genes, including protein farnesyltransferase subunit beta (*FNTB*; 2.5.1.58), STE24 endopeptidase (*STE24*; 3.4.24.84), prenyl protein peptidase (*FACE2*), and protein-S-isoprenylcysteine O-methyltransferase (*STE14*; 2.1.1.100). Among these, *STE24* was significantly up-regulated (unigene9535; CL3149. Contig1; unigene 10944) in male beetles, suggesting that *STE24* may encode a key enzyme in cantharidin biosynthesis.

The fact that both juvenile hormone (JH) and cantharidin can be blocked by 6-fluoromevalonate suggests that cantharidin and JH may be related. However, whether or not JH is the precursor of cantharidin remains unknown [12,14,15]. We identified three genes (cytochrome P450, family 15, subfamily A, polypeptide 1 (*CYP15A1*), JH epoxide hydrolase (*JHEH*) and JH esterase (*JHE*)) from the “insect hormone biosynthesis” pathway (map00981). *CYP15A1* (unigene16612), which encodes cytochrome P450 that catalyzes epoxidation of methyl farnesoate to JHIII in JH biosynthesis [38], shown no differentially expressed between male and female libraries. In insects, the role of JH in adult males is far less than that in females because of the latter’s requirements for oviposition, as same as the gene expression level. Within the two JH degration related genes [39,40], only *JHE* (CL3161.contig1), which catalyzes JH Ш diol to JH Ш acid diol, was down-regulated in the male group. However, interestingly, *JHEH* (unigene18052), which catalyzes JH Ш to JH Ш diol, was up-regulated in males. These results suggest that JH Ш diol may accumulate in adult male beetles and *JHEH* might play an important role in male blister beetles. Whether or not it is related to the cantharidin biosynthesis needs further verification. The candidate genes involved in cantharidin biosynthesis are shown in Table 2.

**Table 2:** Candidate genes involved in cantharidin biosynthesis in *M*. *cichorii*.

Enzyme definition	Enzyme name	Number of unigenes in Illumina transcriptome library
Acetyl-CoA C-acetyltransferase	*atoB*	11
Hydroxymethylglutaryl-CoA synthase	*HMGS*	4
Hydroxymethylglutaryl-CoA reductase	*HMGR*	4
Mevalonate kinase	*mvaK1*	2
Phosphomevalonate kinase	*mvaK2*	2
Diphosphomevalonate decarboxylase	*MVD*	3
Isopentenyl-diphosphate delta-isomerase	*IDI*	2
Farnesyl diphosphate synthase	*FPPS*	6
Protein farnesyltransferase subunit beta	*FNTB*	4
STE24 endopeptidase	*STE24*	4
Prenyl protein peptidase	*FACE2*	5
Protein-S-isoprenylcysteine O-methyltransferase	*STE14*	2
CytochromeP450, family 15, subfamily A, polypeptide 1	*CYP15A1*	1
Juvenile hormone epoxide hydrolase	*JHEH*	4

### RT-qPCR validation of DEGs

**Fig 7:**
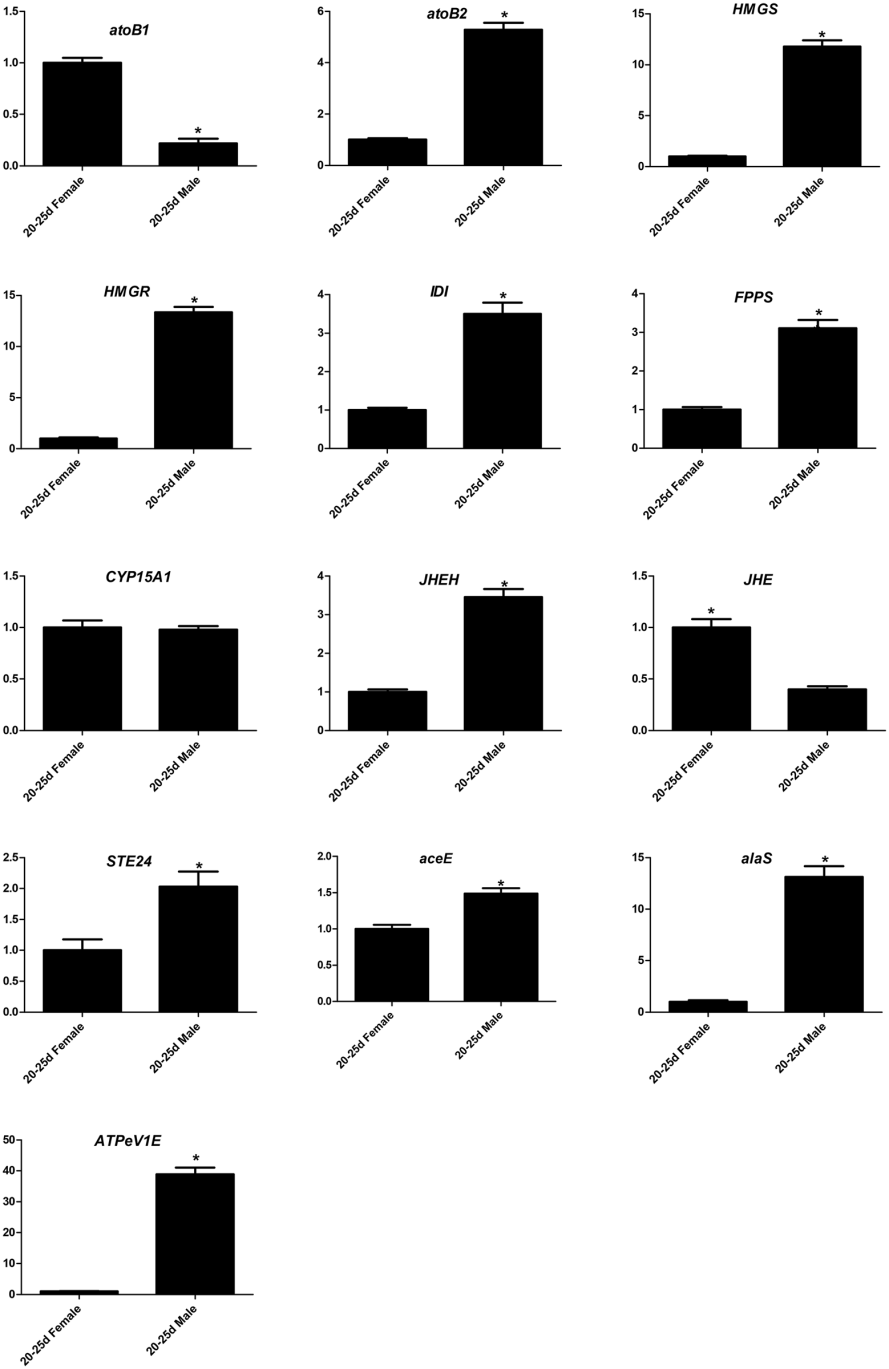
RT-qPCR analysis of 13 unigenes differentially-expressed between 20–25 day-old female and male groups. Line diagrams represent the expression patterns of selected unigenes from the *M*. *cichorii* transcriptome. Expression was compared between female and male groups. Error bars represent standard error. *Significant difference between gene expression (based on 20–25 day-old females, using Student’s *t*-test with p < 0.05).

RT-qPCR was used to confirm the results and expression profiles of the genes identified by Illumina sequencing analysis. We selected 13 unigenes (*atoB1* (unigene5706), *atoB2* (CL951. Contig3), *HMGS* (unigene496), *HMGR* (CL2009. Contig1), *IDI* (CL3032.contig1), *FPPS* (unigene16392), *STE24* (unigene 10944), *CYP15A1* (unigene16612), *JHEH* (unigene 18052), *JHE* (CL3161.contig1), pyruvate dehydrogenase E1 (*aceE*, CL2113.contig2), alanyl-tRNA synthetase (*alaS*, unigene 18541), and V-type H^+^-transporting ATPase subunit E (*ATPeV1E*, unigene 11221)) from specific and typical pathways that were considered to possibly play an important role in cantharidin biosynthesis (Fig 7). Six of these genes (*atoB2*, *HMGS*, *HMGR*, *IDI*, *FPPS*, and *STE24*) were annotated relative to terpenoid backbone biosynthesis and were validated as being highly-expressed in the 20–25 day-old male group (5-, 12-, 13-, 3.5-, 3-, and 2-fold, respectively), while *atoB1* was down-regulated (4-fold) in male. The gene, *CYP15A1*, related to JH biosynthesis shown no differentially expressed between male and female group. The genes (*JHEH* and *JHE*) involved in JH degration were also validated, and indicated that *JHEH* was up-regulated (3.5-fold) while *JHE* was down-regulated (2.5-fold) in the male compared with the female group.

We found that three genes, *aceE*, *alaS*, and *ATPeV1E*, related to the tricarboxylic acid cycle, aminoacyl-tRNA biosynthesis, and oxidative phosphorylation (all involved in material and energy metabolism pathways) displayed higher expression in male compared to female beetles (1.5-, 13-, and 39-fold, respectively). This suggested the generation of secondary metabolites was active and inevitably associated with the changes in substance circulation and energy flow.

These results were consistent with the results of Illumina sequencing, which therefore validated the expression profiles of the genes and also verified the reliability and accuracy of our transcriptome analysis.

## Conclusions

In this study, we investigated the *de novo* transcriptome and expression profile of *M*. *cichorii* using Illumina deep-sequencing technology. We identified 2,465 unigenes that were significantly differentially-expressed between adult male and female beetles. Only unigenes involved in the MVA pathway, including all the necessary enzymes, were found in the transcriptome databases of *M*. *cichorii*, confirming that cantharidin biosynthesis in *M*. *cichorii* most likely occurs via the MVA pathway, not the MEP pathway or a mixture of these. In addition, we also found that cantharidin biosynthesis might be related to the juvenile hormone (JH) biosynthesis or degradation. Globally-identified cantharidin biosynthesis-related genes and putative biosynthesis pathways in *M*.*cichorii* suggests that cantharidin biosynthesis may be more complicated than previously believed. The large number of transcripts obtained in this study will provide a strong basis for future research into the genomics, gene expression, and functional genomics of the blister beetle *M*. *cichorii*.

## Supporting Information

S1 Fig Histogram presentation of differentially-expressed gene GO classification. (PDF)

S1 Table Primers used for RT-qPCR analysis. (PDF)

S2 Table Top ten differentially-expressed genes between the male and female libraries. (PDF)
