# Supplementary material for: De Novo Transcriptome and Expression Profile Analysis to Reveal Genes and Pathways Potentially Involved in Cantharidin Biosynthesis in the Blister Beetle Mylabris cichorii
Source: PLoS One. 2016 Jan 11;11(1):e0146953. doi: 10.1371/journal.pone.0146953 (PMC4709229; doi:10.1371/journal.pone.0146953)

S1 Figure. Histogram presentation of differentially expressed gene GO classification. The results are summarized in three main categories: biological process, cellular component and molecular function. The right y-axis indicates the number of genes in a category. The left y-axis indicates the percentage of a specific category of genes in that main category.

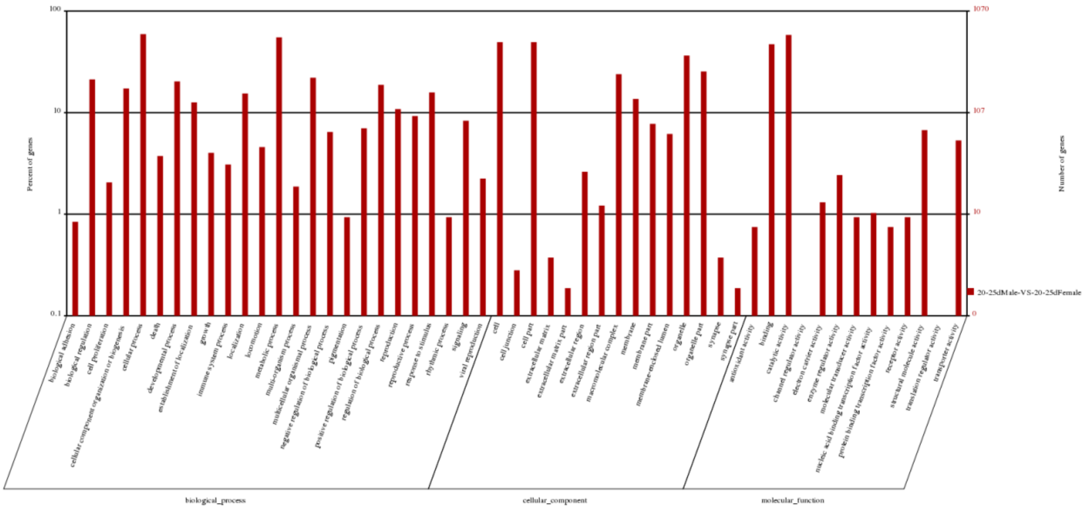

Supplement: S1 Fig — (PDF) [file pone.0146953.s001.pdf]
