# Supplementary material for: De Novo Transcriptome and Expression Profile Analysis to Reveal Genes and Pathways Potentially Involved in Cantharidin Biosynthesis in the Blister Beetle Mylabris cichorii
Source: PLoS One. 2016 Jan 11;11(1):e0146953. doi: 10.1371/journal.pone.0146953 (PMC4709229; doi:10.1371/journal.pone.0146953)
Supplement: S1 Table — (PDF) [file pone.0146953.s002.pdf]

S1 Table. Primers used for RT-qPCR analysis

Gene	Primer sequences (forward and reverse)
RPL22e	5'-AGGCTTGAAGAAGAAGAAG-3'<br>5'-ATGTTGGCTGGATTTACC-3'
UBE3A	5'-TTAGTGAATGCTCTAGTAAC-3'<br>5'-CGGTAATGCTGTCTCTAA-3'
atoB1	5'-TGAAGGATGGCTTGACAGAC-3'<br>5'-GTGCGAATGGCATAATCATC-3'
atoB2	5'-AACTCCCGGTAATGCATCTG-3'<br>5'-GCCAACGGTTTGAGATTCTT-3'
HMGS	5'-AACCGATAAGGACGTTACGC-3'<br>5'-GAAGCCCATCTTCTCCTGTC-3'
HMGR	5'-GAAGGTCGTGGTAAATCGGT-3'<br>5'-GCAACAGCTGAACCAATCAT-3'
IDI	5'-GCGAGGATAACGAGGAGAAC-3'<br>5'-GCCATTCCCTTCGTCTTTAT-3'
FPPS	5'-AAGCCCGAATTACACGAAAC-3'<br>5'-TCGATGCGATATTTGGTGAT-3'
STE24	5'-CCACACAATTGAATTTCCCA-3'<br>5'-CCACACAAATAGGCGTTACTG-3'
CYP15A1	5'-GGCGACTTGTGGTCTATTCA-3'<br>5'-CTTCACCATCAATTCCATCG-3'
JHEH	5'-CGAACATGTGTTATGCGACA-3'<br>5'-CAAGAAGACGATTGGCGTAA-3'
JHE	5'-GCAATTTGAGCGGTCTAACA-3'<br>5'-ATTGAAACGACTCACCCACA-3'
aceE	5'-ATGAAACGGCACAAATTGAA-3'<br>5'-ACCGTGCTTGAAATTCCTTC-3'
alaS	5'-GGAAGTTGTAGGGACCGATG-3'<br>5'-TTCGTCAACAAATTCCAAGC-3'
ATPeV1E	5'-CGTATCACCCAAATGATTGC-3'<br>5'-AAACGTTCCTCTTGGCAAAT-3'
