# Supplementary material for: De Novo Transcriptome and Expression Profile Analysis to Reveal Genes and Pathways Potentially Involved in Cantharidin Biosynthesis in the Blister Beetle Mylabris cichorii
Source: PLoS One. 2016 Jan 11;11(1):e0146953. doi: 10.1371/journal.pone.0146953 (PMC4709229; doi:10.1371/journal.pone.0146953)
Supplement: S2 Table — (PDF) [file pone.0146953.s003.pdf]

S2 Table. Top ten differentially-expressed genes between the male and female libraries.

Unigene ID	Hit Number	Discription	FDR	Log2Ratio (20-25dMale/20-25dFemale)
CL46.contig2[a]			0	17.63
Unigene10092[a]	gi|131093|sp|P17502.1|	RecName: Full=Protamine	0	17.02
Unigene12361[a]			0	16.72
Unigene3713[a]			0	16.53
Unigene16134[a]			5.09E-226	16.08
Unigene15385[a]	gi|340729244|ref|XP_003402916.1|	PREDICTED: trypsin-7-like [*Bombus terrestris*]	0	15.57
Unigene10584[a]			2.71E-178	15.29
Unigene13807[a]	gi|189235559|ref|XP_968512.2|	similar to CG17377 CG17377-PA [*Tribolium castaneum*]	0	15.15
CL2694.contig1[a]	gi|307168676|gb|EFN61712.1|	Trypsin-1 [*Camponotus floridanus*]	0	14.95
Unigene6261[a]			1.82E-193	14.93
Unigene2879[b]			0	-16.73
Unigene9815[b]			1.86E-163	-14.72
Unigene2890[b]			0	-14.71
Unigene11733[b]			2.91E-263	-14.55
CL235.contig3[b]	gi|263191198|ref|NP_001161084.1|	chymotrypsin-like proteinase 6D precursor [*Tribolium castaneum*]	1.27E-212	-14.16
Unigene3672[b]	gi|186396|gb|AAA59163.1|	mucin, partial [*Homo sapiens*]	6.11E-174	-14.12
Unigene3792[b]	gi|325459340|gb|ADZ13687.1|	secretory eggshell protein precursor [*Clonorchis sinensis*]	2.78E-153	-13.40
Unigene6643[b]	gi|471403978|ref|XP_004384164.1|	PREDICTED: protein PRRC2C [*Trichechus manatus latirostris*]	2.05E-100	-13.19
Unigene10980[b]			5.21E-146	-13.17
CL2856.contig1[b]	gi|289191335|ref|NP_001166054.1|	serine protease 120 precursor [*Nasonia vitripennis*]	1.73E-95	-13.17

The corner marks of gene ID: ‘a’ represents up-regulated genes and ‘b’ represents down-regulated genes.
